# Identification of patients with unstable angina based on coronary CT angiography: the application of pericoronary adipose tissue radiomics

**DOI:** 10.3389/fcvm.2024.1462566

**Published:** 2024-12-12

**Authors:** Weisheng Zhan, Yixin Li, Hui Luo, Jiang He, Jiao Long, Yang Xu, Ying Yang

**Affiliations:** ^1^ Cardiovascular Medicine Department, Affiliated Hospital of North Sichuan Medical College, Nanchong, China; ^2^ Digestive System Department, Affiliated Hospital of North Sichuan Medical College, Nanchong, China; ^3^ Thoracic Surgery Department, Nan Chong Center Hospital, Nanchong, China; ^4^ Dermatological Department, Nan Chong Center Hospital, Nanchong, China

**Keywords:** pericoronary adipose tissue, radiomics, coronary computed tomography angiography, coronary heart disease, machine learning

## Abstract

**Objective:**

To explore whether radiomics analysis of pericoronary adipose tissue (PCAT) captured by coronary computed tomography angiography (CCTA) could discriminate unstable angina (UA) from stable angina (SA).

**Methods:**

In this single-center retrospective case-control study, coronary CT images and clinical data from 240 angina patients were collected and analyzed. Patients with unstable angina (*n* = 120) were well-matched with those having stable angina (*n* = 120). All patients were randomly divided into training (70%) and testing (30%) datasets. Automatic segmentation was performed on the pericoronary adipose tissue surrounding the proximal segments of the left anterior descending artery (LAD), left circumflex coronary artery (LCX), and right coronary artery (RCA). Corresponding radiomic features were extracted and selected, and the fat attenuation index (FAI) for these three vessels was quantified. Machine learning techniques were employed to construct the FAI and radiomic models. Multivariate logistic regression analysis was used to identify the most relevant clinical features, which were then combined with radiomic features to create clinical and integrated models. The performance of different models was compared in terms of area under the curve (AUC), calibration, clinical utility, and sensitivity.

**Results:**

In both training and validation cohorts, the integrated model (AUC = 0.87, 0.74) demonstrated superior discriminatory ability compared to the FAI model (AUC = 0.68, 0.51), clinical feature model (AUC = 0.84, 0.67), and radiomic model (AUC = 0.85, 0.73). The nomogram derived from the combined radiomic and clinical features exhibited excellent performance in diagnosing and predicting unstable angina. Calibration curves showed good fit for all four machine learning models. Decision curve analysis indicated that the integrated model provided better clinical benefit than the other three models.

**Conclusions:**

CCTA-based radiomics signature of PCAT is better than the FAI model in identifying unstable angina and stable angina. The integrated model constructed by combining radiomics and clinical features could further improve the diagnosis and differentiation ability of unstable angina.

## 1 Introduction

Coronary artery disease (CAD), predominantly caused by atherosclerosis, is the leading cause of cardiovascular mortality, posing a significant threat to human life worldwide (1, 2). Recent statistics indicate that individuals suffering from angina pectoris constitute half of the CAD patient population. Stable angina and unstable angina are the two principal forms of this condition, primarily manifesting clinically as chest pain of varying degrees (3).

Patients with unstable angina are at a higher risk of major adverse cardiovascular events (MACE) and mortality compared to those with stable angina (4). Moreover, when selecting different treatment strategies for stable angina and unstable angina, SA patients undergoing percutaneous coronary intervention (PCI) do not exhibit a significant difference in long-term MACE occurrence compared to those receiving medication therapy (5, 6). In contrast, UA patients require rapid assessment and management before deciding on PCI treatment (7). Therefore, the early and rapid diagnosis and differentiation of these conditions are crucial. Clinically, experienced medical personnel often make preliminary diagnoses based on the severity, frequency, and duration of the patient's chest pain (8). These initial assessments are then further evaluated with electrocardiograms, laboratory results, and echocardiographic parameters. However, these symptoms and tests lack specificity, and there is a deficiency of effective biomarkers for the diagnosis of unstable angina (9, 10). Coronary CT angiography (CTA) can measure and assess vascular calcification and narrowing, possessing an excellent negative predictive value, making it the current first-line imaging tool for diagnosing unstable angina (11). Coronary artery wall inflammation is a key factor leading to the instability of atherosclerotic plaques, promoting plaque progression and rupture, exacerbating luminal stenosis, and triggering severe cardiovascular diseases such as acute coronary syndrome (ACS) (12). Recent evidence suggests that during the atherosclerotic process, inflammatory vasculature secretes a variety of pro-inflammatory factors into the pericoronary adipose tissue through paracrine mechanisms, leading to dynamic changes in lipid-water balance. These changes can be captured on CCTA via the fat attenuation index (13). FAI quantifies alterations in PCAT composition caused by vascular inflammation by recording CT density attenuation changes. It is a sensitive biomarker that dynamically reflects coronary inflammation (13, 14). However, vascular inflammation not only causes changes in the FAI value through increased lipid breakdown, decreased lipogenesis, and intracellular edema in PCAT but also leads to more persistent changes in the perivascular space, such as fibrosis and neovascularization (15, 16). These persistent adverse structural changes are beyond the measurement capabilities of FAI. Nevertheless, with the application of artificial intelligence in medicine, emerging technologies such as radiomics can overcome the limitations of subjective human visual analysis. Radiomics allows for the non-invasive extraction of vast databases of rich radiomic features from medical images, enabling comprehensive, objective, and quantitative assessment of lesion heterogeneity (17).

Radiomics is a novel technique that extracts thousands of quantitative features from medical images and uses methods such as machine learning to identify the most valuable imaging features (18). Its application in oncology and other fields is already well-established, providing diagnostic assessment and clinical decision support for cancer patients (15, 17).

In recent years, pericoronary adipose tissue (PCAT) has emerged as a significant focus of research in the field of cardiovascular diseases. This is largely due to the accumulating evidence suggesting that the characteristics of PCAT, such as its density and texture, may have a substantial relationship with the presence and severity of coronary artery lesions. Understanding these relationships is crucial, as they can potentially lead to new diagnostic and therapeutic approaches for coronary artery disease (19–21). Unstable angina (UA) and stable angina (SA) represent two distinct clinical presentations of coronary artery disease, each with different prognostic implications and management strategies. Distinguishing between these two types is critical, as misclassification can lead to inappropriate treatment and increased risk of adverse cardiovascular events. Despite advancements in imaging techniques, effectively differentiating UA from SA remains a significant challenge for clinicians.

Existing literature has explored various imaging modalities and biomarkers for distinguishing UA from SA; however, few studies have utilized radiomics to analyze PCAT specifically. While some studies have highlighted the potential of radiomics in cardiovascular imaging, they often focus on different anatomical regions or disease states (22–24). Our approach is unique in that it specifically applies radiomics analysis to PCAT, utilizing coronary computed tomography angiography (CCTA) as the imaging modality. The primary objective of this study is to evaluate whether radiomics analysis of PCAT, as captured by coronary computed tomography angiography (CCTA), can effectively differentiate UA from SA. By employing advanced imaging and analytical techniques, we aim to provide insights into the diagnostic capabilities of PCAT radiomics (25–27). Clearly articulating this objective not only highlights the rationale behind our research but also underscores its potential impact on improving clinical decision-making and patient outcomes.

## 2 Materials and methods

### 2.1 Study population and data collection

**Figure 1:**
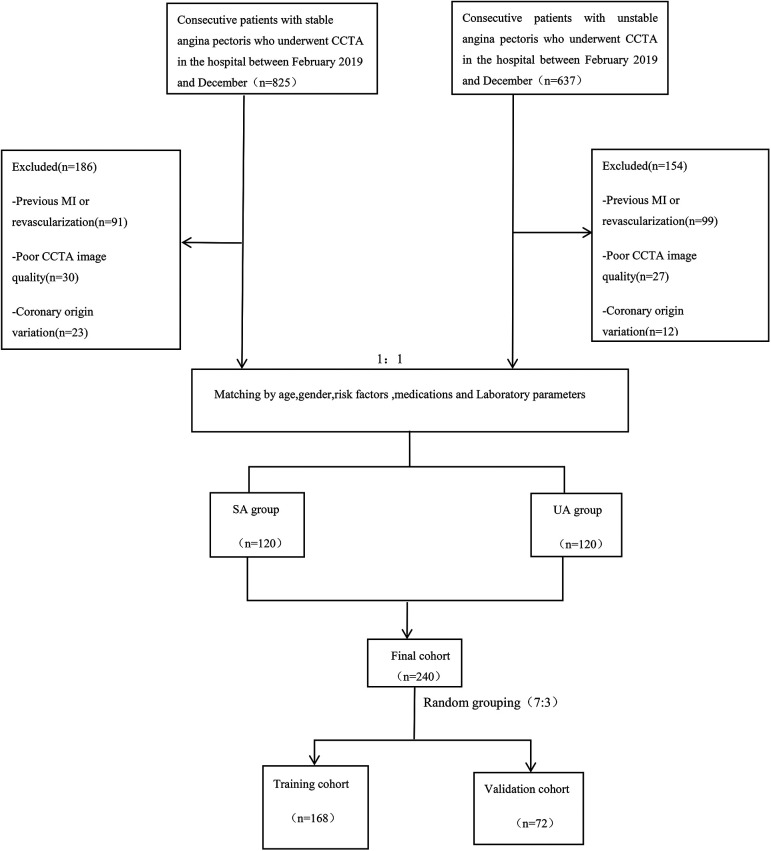
Flow chart showing inclusion and exclusion criteria for the study population. CCTA, coronary computed tomography angiography; SA, stable angina; UA, unstable angina; MI, myocardial infarction.

This retrospective single-center study was approved by the Medical Ethics Committee of the Affiliated Hospital of North Sichuan Medical College (Approval No.: 2023ER218-1). The entire experimental process adhered to the principle of informed immunity. The study is in accordance with the Declaration of Helsinki. We systematically collected data from 240 patients with angina pectoris who attended the Cardiac Center of the Affiliated Hospital of North Sichuan Medical College and underwent CCTA from February 2019 to December 2023. The cohort included 120 patients with stable angina and 120 with unstable angina [UA patients met the diagnostic criteria for UA according to the 2021 American Heart Association/American College of Cardiology/American Society of Echocardiography/CHEST/Society for Academic Emergency Medicine/Society of Cardiovascular Computed Tomography/Society for Cardiovascular Magnetic Resonance chest pain evaluation and management guidelines (5), and SA patients conformed to the 2019 European Society of Cardiology guidelines for the diagnosis of chronic coronary syndromes (28)]. Exclusion criteria included a history of myocardial infarction or revascularization, poor CCTA image quality, anomalies in coronary artery origin or termination, and incomplete medical records. A detailed flowchart outlining the patient selection process and study design is provided in Figure 1. Additionally, we collected relevant clinical history data for all patients enrolled in the study, including demographic characteristics (gender, age, and body mass index), cardiovascular risk factors (smoking, hypertension, diabetes, hyperlipidemia, and medication history), laboratory parameters, and echocardiographic indicators. Informed consent was obtained from all patients prior to their participation in the study. Furthermore, patient data were anonymized to protect their privacy and confidentiality throughout the research process.

### 2.2 CCTA acquisition

All subjects selected for the study underwent scanning with a dual-source 2*96 detector row CT scanner (Stellar Infinity detector, SOMATOM Force, Germany). The scanner utilized prospective electrocardiogram gating with tube voltages ranging from 70 to 150 kV, a rotation speed of 0.25 s, and a temporal resolution of 66 ms per segment, making it capable of accommodating free heart rates during coronary CT. It also featured a holistically designed photon detector with Edge and Ultra-High Resolution (UHR) technology, reducing electronic crosstalk between adjacent detectors, allowing the reconstruction of 0.6 mm acquisition slices into 0.4 mm thin slices. To minimize artifacts caused by respiratory motion, each patient underwent breath-holding training prior to scanning, and all extraneous objects that could degrade image quality were removed. Scans were performed in a breath-hold state, with the upper boundary at the thoracic inlet and the lower edge down to 1 cm below the cardiac diaphragmatic surface; the CCTA data acquisition range started 2 cm below the tracheal carina down to 1 cm below the cardiac diaphragmatic surface, with the left and right boundaries extending 1–2 cm beyond the heart margin.

### 2.3 Quantification measurement of FAI

Pericoronary adipose tissue was defined as all voxels within a radial distance from the vessel wall equal to the diameter of the vessel, within the range of −190 to −30 Hounsfield Units (HU). The Fat Attenuation Indexwas derived by calculating the average attenuation value of the pericoronary adipose tissue (i.e., the mean CT value of PCAT). To comprehensively assess and monitor the impact of inflammation on the coronary arteries, the regions of interest (ROIs) for the three most significant epicardial vessels - RCA, LAD, and LCX - were all automatically measured using software in this study. To ensure the standardization and reproducibility of data collection, FAI measurements for all participants were performed using intelligent quantification by Shukun Technology Co., Ltd, Beijing, China, Version:6.21.

### 2.4 PCAT segmentation and radiomics feature extraction

**Figure 2:**
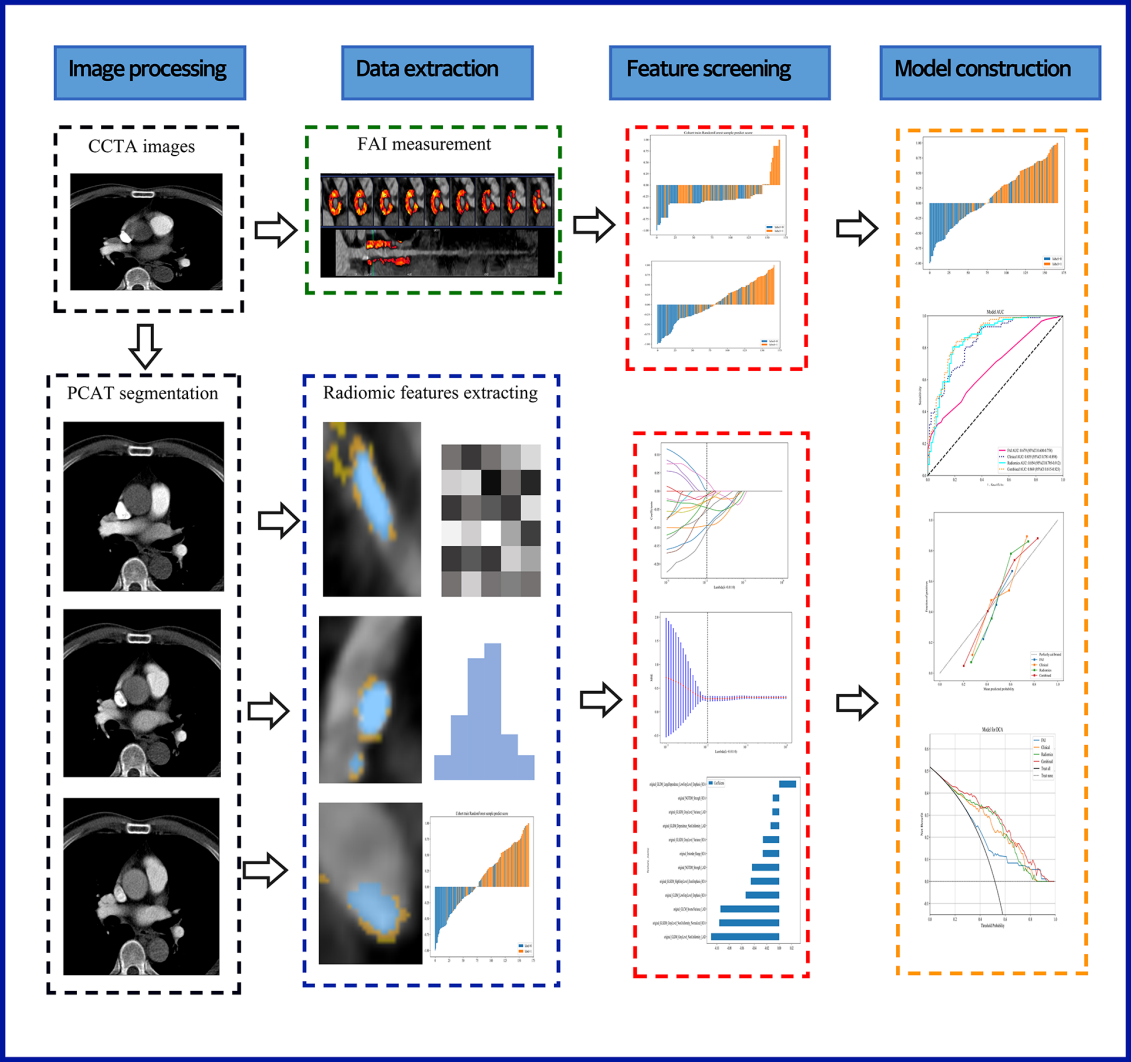
A flow chart displaying the process for development of radiomicsbased integrated model. CCTA, coronary computed tomography angiography; PCAT, pericoronary adipose tissue; FAI, fat attenuation index.

Recent studies indicate that PCAT is a sensitive imaging biomarker for the vulnerability of plaques surrounding the coronary arteries (25). Therefore, in our study, we performed PCAT segmentation on all three of the most significant epicardial vessels. Using Shukun Technology software (14), which employs a trained deep learning model for coronary segmentation and a contracting skeleton algorithm to calculate the centerline of each coronary artery, we automatically tracked a 40 mm segment of interest in the proximal sections of the LAD, LCX, and RCA. For the LAD and LCX, a 40 mm section following the bifurcation of the left main coronary artery was analyzed. For the RCA, to avoid the influence of the aortic wall, we excluded the proximal 10 mm segment and focused on the segment from 10 mm downstream of the aortic root to 50 mm from the proximal end of the RCA. Additionally, when segmenting coronary plaques in the corresponding regions, we set the cross-sectional area of the segmentation to three times the diameter of the vessel lumen to further ensure data comprehensiveness. A total of 94 radiomic features were extracted from the PCAT surrounding each coronary plaque, including morphological features, first-order histogram features, and higher-order texture features, yielding a total of 94*3 radiomic features per patient. Figure 2 illustrates the radiomics workflow utilized in this study.

### 2.5 Feature selection and prediction model building

Random Forest was used as the machine learning method to build the models for differentiating unstable angina (UA) from stable angina (SA). This algorithm was chosen due to its robustness and ability to handle high-dimensional data, which is a common characteristic in radiomics studies. Random Forest is less prone to overfitting compared to other algorithms, such as support vector machines (SVM) or neural networks, especially when dealing with small sample sizes typical in medical research.

Additionally, Random Forest provides inherent feature importance, allowing us to identify the most relevant imaging features contributing to the model's predictions. This interpretability is particularly beneficial in a clinical context where understanding the basis for decision-making is essential.

For hyperparameter optimization, we employed a grid search method combined with cross-validation to systematically explore different parameter settings. Key hyperparameters such as the number of trees and the maximum depth of the trees were optimized to enhance model performance.

The performance of the Random Forest model was evaluated using several metrics beyond cross-validation, including accuracy, precision, recall, and the area under the receiver operating characteristic curve (AUC-ROC). These metrics provide a comprehensive assessment of the model's predictive capability, ensuring its applicability in clinical decision-making.

This experiment employed machine learning methods (Random Forest) to establish four models, comparing and analyzing each model's clinical performance in diagnosing and differentiating between stable angina and unstable angina. The detailed model-building process is as follows:

#### 2.5.1 FAI model

FAI values for the three critical coronary vessels were included in our study, but due to the relatively small number of input features, we directly developed a machine learning model.

#### 2.5.2 Clinical model

We collected and organized as many clinical predictors used to differentiate between UA and SA as possible, including patient baseline conditions, cardiovascular risk factors, laboratory indicators, and echocardiographic parameters. First, univariate logistic regression was used to filter predictors with *P* < 0.05. These clinical variables were then subjected to multivariate analysis, resulting in the identification of the most relevant clinical features. Using machine learning (Random Forest), we constructed an independent clinical model based on these predictors.

#### 2.5.3 Radiomics model

Initially, *z*-score standardization was used to minimize scale differences between various radiomic features, normalizing the raw imaging data. We performed a Mann-Whitney *U* test on all radiomic features, retaining only those with *P* < 0.05. For features with high redundancy, we employed Spearman correlation analysis and excluded those with a correlation coefficient >0.9. Subsequently, the remaining radiomic features underwent further selection validation using the Least Absolute Shrinkage and Selection Operator (LASSO) algorithm, resulting in the final radiomic feature values with strong stability and high relevance. Ultimately, these selected radiomic feature values were input into machine learning (Random Forest) to construct the corresponding radiomics model.

#### 2.5.4 Integrated model

We combined the independently selected clinical features with the integrated radiomic features to build a comprehensive machine learning model. Based on this model, a corresponding comprehensive nomogram was developed. To prevent overfitting and balance the limited sample size, all four ML models were calibrated using a 10-fold cross-validation approach (29).

### 2.6 Statistical analysis

The analysis was conducted using Python, specifically employing the following libraries:NumPy (version 1.21.0) for numerical computations; pandas (version 1.3.0) for data manipulation and analysis; scikit-learn (version 0.24.2) for machine learning and model building; statsmodels (version 0.12.2) for statistical modeling and hypothesis testing; matplotlib (version 3.4.2) for data visualization. For continuous (quantitative) data, the Shapiro-Wilk test was utilized to determine normality; if normally distributed, means ± standard deviations (*X* ± *S*) were used, and comparisons between two groups were made using independent sample *t*-tests; if not normally distributed, medians (25th percentile, 75th percentile) were represented, with comparisons between groups made using the Wilcoxon test. For analyzing categorical data, the *χ*^2^ test was employed unless the expected frequency in any category was less than 5, in which case Fisher's exact test was utilized. This approach ensures that the assumptions of the tests are met, enhancing the reliability of the results. A two-sided *P*-value less than 0.05 was considered statistically significant. Calibration curves demonstrated good agreement between predicted probabilities and observed outcomes, with a calibration slope of 0.95 and an intercept of −0.02, indicating minimal systematic bias. These thresholds provide a quantitative measure of what constitutes “good” agreement. Decision curve analysis (DCA) was performed to assess the net benefit of the predictive model. The net benefit was quantified by calculating the difference between true positive rates and the weighted false positive rates across a range of threshold probabilities. This analysis illustrates the clinical utility of the model by demonstrating how its implementation could influence decision-making in practice. A power analysis was conducted using G*Power software to determine the appropriate sample size for the study. Assuming a medium effect size (Cohen's *d* = 0.5) based on preliminary data, with a significance level set at 0.05 and a desired power of 80%, the analysis indicated that a total sample size of 240 patients would be sufficient to detect statistically significant differences between unstable angina (UA) and stable angina (SA). This sample size is considered adequate to ensure the reliability of the statistical tests employed in the analysis.

## 3 Results

### 3.1 General baseline data

All included participants were randomly divided into a training dataset (*n* = 168) and a testing dataset (*n* = 72) in a 7:3 ratio. Table 1 also recorded the clinical baseline characteristics of the 240 study subjects in both the training and testing sets. Statistical tests revealed significant differences (*P* < 0.05) between cases of stable angina and unstable angina in terms of age, LDL-C (Low-Density Lipoprotein Cholesterol), BMI (Body Mass Index), radiation dose, FS (fractional shortening), and MVA (mitral valve area), while the remaining indicators showed no significant statistical differences. The balance of stable and unstable angina groups across the training and testing datasets indicates good patient characteristic matching.

**Table 1:** Baseline characteristics of the study population.

Characteristics	Total (*n* = 240)	SA group (*n* = 120)	UA group (*n* = 120)	*P* value
Clinical characteristics				
Age	64.10 ± 10.73	61.97 ± 10.50	66.22 ± 10.57	0.002
Smoking	0.37 ± 0.48	0.35 ± 0.48	0.39 ± 0.49	0.453
BMI	25.19 ± 2.70	24.70 ± 2.54	25.67 ± 2.78	0.008
Male gender	130 (54.17)	63 (52.50)	67 (55.83)	0.698
Hypertension	138 (57.50)	65 (54.17)	73 (60.83)	0.361
Diabetes	45 (18.75)	19 (15.83)	26 (21.67)	0.321
Baseline medications				
Antiplatelet	44 (18.33)	21 (17.50)	23 (19.17)	0.868
Beta-blocker	16 (6.67)	9 (7.50)	7 (5.83)	0.796
ACEI/ARB	26 (10.83)	13 (10.83)	13 (10.83)	1.000
Statin	0.18 ± 0.38	0.18 ± 0.38	0.17 ± 0.38	0.891
Lipids, mmol/L				
Triglycerides	1.70 ± 1.00	1.63 ± 0.90	1.76 ± 1.09	0.358
Total-cholesterol	4.57 ± 1.20	4.67 ± 1.20	4.48 ± 1.20	0.210
LDL	3.13 ± 1.39	3.47 ± 1.56	2.80 ± 1.09	<0.001
HDL	1.16 ± 0.47	1.20 ± 0.59	1.12 ± 0.32	0.777
Inflammatory markers				
White cell count, ×10^9^/L	6.83 ± 1.70	6.73 ± 1.73	6.93 ± 1.67	0.238
CCTA acquisition parameters				
Radiation dose, DLP	412.67 ± 276.49	382.13 ± 256.39	443.21 ± 293.13	0.039
Tube voltage 70 kv	134 (55.83)	66 (55.00)	68 (56.67)	0.897
80 kv	89 (37.08)	46 (38.33)	43 (35.83)	0.789
90 kv	10 (4.17)	5 (4.17)	5 (4.17)	1.000
110 kv	7 (2.92)	3 (2.50)	4 (3.33)	1.000
Heart rate	74.08 ± 10.71	74.34 ± 9.86	73.82 ± 11.53	0.427
Systolic_pressure	133.74 ± 23.61	135.07 ± 25.76	132.40 ± 21.27	0.697
Diastolic_pressure	76.53 ± 10.36	77.02 ± 10.34	76.03 ± 10.40	0.463
Ultrasonic cardiogram				
LVDD	46.17 ± 4.63	46.30 ± 4.79	46.04 ± 4.48	0.727
LAD	35.08 ± 5.23	34.77 ± 3.87	35.39 ± 6.31	0.63
RAD	42.55 ± 19.70	43.92 ± 27.35	41.17 ± 5.29	0.054
RVDD	21.69 ± 2.32	21.70 ± 2.01	21.68 ± 2.60	0.339
EF	0.63 ± 0.07	0.64 ± 0.06	0.63 ± 0.07	0.768
FS	0.50 ± 1.85	0.63 ± 2.61	0.36 ± 0.07	0.009
EDV	42.05 ± 28.47	40.84 ± 25.61	43.26 ± 31.12	0.812
MVE	0.72 ± 0.18	0.71 ± 0.15	0.73 ± 0.20	0.836
MVA	0.89 ± 0.18	0.87 ± 0.17	0.92 ± 0.19	0.04

*P* values were derived from the univariable association analysis between different variables; data are means with a statistical difference. *P* value reflected the differences between the SA cohort and UA cohort. SA, stable angina; UA, unstable angina; LDL, low-density lipoprotein; HDL, high-density lipoprotein; CCTA, coronary computed tomography angiography; DLP, dose-length product; BP, blood pressure; BMI, body mass index; ACEI, angiotensin converting enzyme inhibitor; ARB, vasopressin II receptor blocker; LVDD, left atrium end diastolic diameter; LAD, left atrium diameter; RAD, right atrium diameter; RVDD, right ventricular end diastolic diameter; EF, ejection fraction; FS, fraction shorting; EDV, end-diastolic volume; MVE, mitral valve echogram; MVA, mitral valve area.

### 3.2 Feature selection and prediction model building

**Figure 3:**
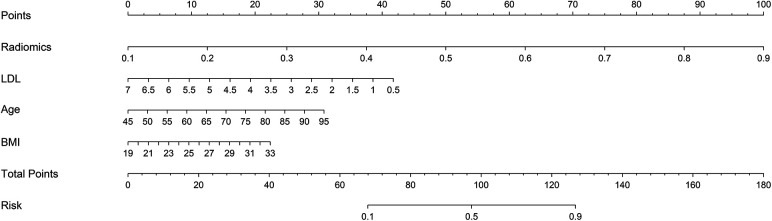
Developed combined model nomogram. The combined score nomogram was develpoed in the training dataset with low-density lipoprotein (LDL), body mass index (BMI), age and radiomics.

In this study, 94 pericoronary adipose tissue radiomic features were automatically extracted from the proximal LAD, LCX, and RCA in both the training and testing cohorts (a total of 282 features). These features underwent Mann-Whitney *U* test filtering, retaining 40 features with *P* < 0.05. Redundancy was eliminated through Spearman correlation analysis, leaving 18 radiomic features. Dimensionality reduction was then performed on the remaining features using LASSO regression, resulting in 12 optimal radiomic features (including 1 first-order histogram feature, 1 s-order morphological feature, and 10 higher-order texture features). These 12 optimal features were used to construct the radiomics model. Figure 2 illustrates the selection process for the study's radiomic features. The same process was used to construct the FAI model using the extracted fat attenuation index values. All collected independent clinical predictive factors, after univariate and multivariate logistic regression analysis, showed that age, LDL, and BMI could serve as independent clinical factors for diagnosing and distinguishing between stable and unstable angina (*P* < 0.05). Based on these 3 clinical factors, a clinical model was constructed using machine learning methods. Detailed results are provided in Table 2. Following the analysis, we integrated clinical risk factors with radiomic features to form 4 independent influencing factors, which were used to build a combined nomogram model (see Figure 3). The nomogram allows for scoring of each independent variable according to a scale, with the total score indicating the likelihood and accuracy of diagnosing unstable angina.

**Table 2:** Logical regression analysis of the clinical independent predictors.

Variables	Univariate regression analysis			Multivariate regression analysis		
	OR	95% CI	*P*	OR	95% CI	*P*
Age	1.010	1.004, 1.016	0.005	1.011	1.005, 1.016	0.002
Gender	1.070	0.941, 1.218	0.386	-	-	-
Body mass index	1.035	1.011, 1.060	0.017	1.039	1.016, 1.063	0.006
Hypertension	1.137	1.000, 1.294	0.101	-	-	-
Diabetes	1.105	0.935, 1.306	0.323	-	-	-
Smoking	1.028	0.899, 1.175	0.733	-	-	-
Triglycerides	1.031	0.965, 1.102	0.445	-	-	-
Total cholesterol	0.987	0.935, 1.041	0.680	-	-	-
LDL cholesterol	0.907	0.867, 0.948	0.000	0.913	0.875, 0.954	0.001
HDL cholesterol	1.033	0.881, 1.212	0.739	-	-	-
Antiplatelet	0.958	0.804, 1.140	0.682	-	-	-
Statin	0.979	0.820, 1.169	0.844	-	-	-
Beta-blocker	1.119	0.829, 1.511	0.537	-	-	-
ACE-I or ARB	1.043	0.848, 1.284	0.737	-	-	-
White cell count	1.041	1.001, 1.082	0.093	-	-	-
Systolic BP	1.000	0.997, 1.002	0.844	-	-	-
Diastolic BP	1.000	0.993, 1.006	0.913	-	-	-
Heart rate	0.999	0.993, 1.005	0.745	-	-	-
LVDD	0.993	0.980, 1.006	0.389	-	-	-
LAD	0.999	0.987, 1.012	0.900	-	-	-
RAD	0.998	0.995, 1.001	0.279	-	-	-
RVDD	0.994	0.968, 1.021	0.711	-	-	-
EF	0.614	0.242, 1.559	0.388	-	-	-
FS	0.981	0.952, 1.009	0.267	-	-	-
EDV	1.001	0.999, 1.003	0.510	-	-	-
MVE	1.067	0.748, 1.523	0.763	-	-	-

LDL, low-density lipoprotein; HDL, high-density lipoprotein; CCTA, coronary computed tomography angiography; BP, blood pressure; BMI, body mass index; ACEI, angiotensin converting enzyme inhibitor; ARB, vasopressin II receptor blocker; LVDD, left atrium end diastolic diameter; LAD, left atrium diameter; RAD, right atrium diameter; RVDD, right ventricular end diastolic diameter; EF, ejection fraction; FS, fraction shorting; EDV, end-diastolic volume; MVE, mitral valve echogram.

### 3.3 Performance evaluation

**Figure 4:**
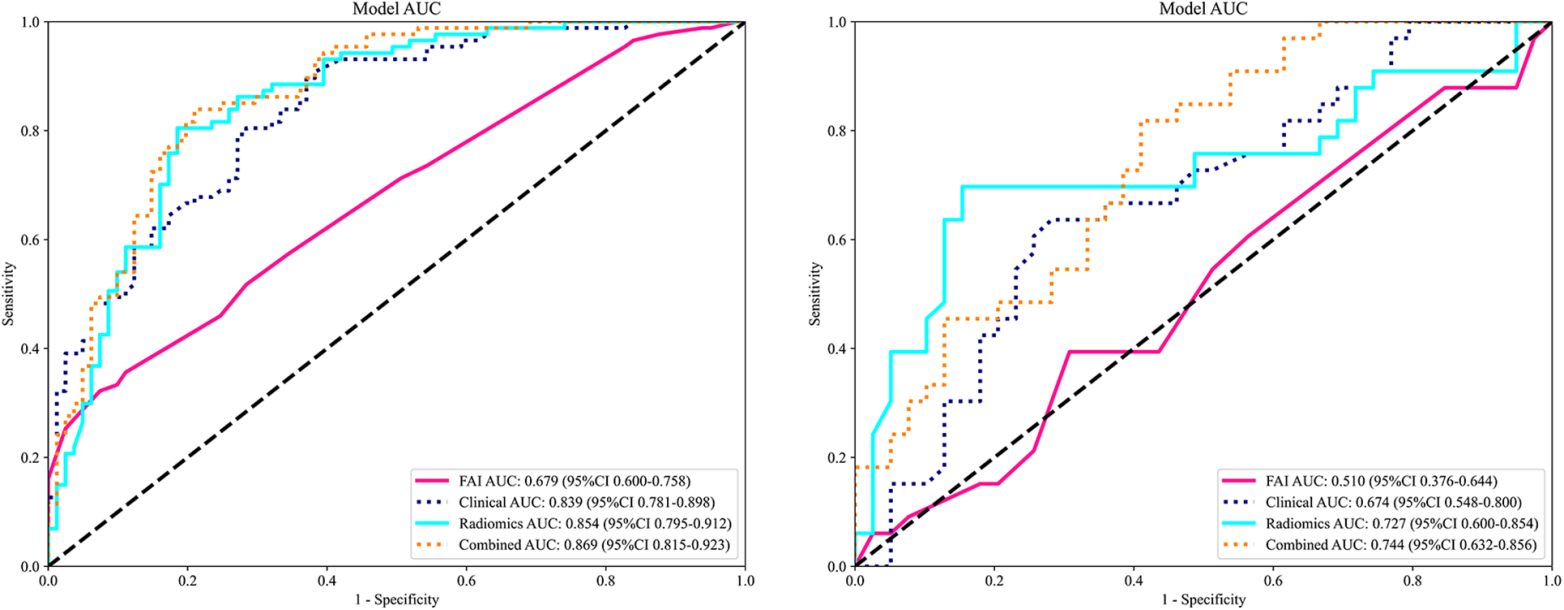
Comparison of receiver operating characteristic (ROC) curves for the FAI model (red lines), clinical model (blue dotted lines), radiomics model (blue solid lines) and combined model (yellow dotted lines).

**Figure 5:**
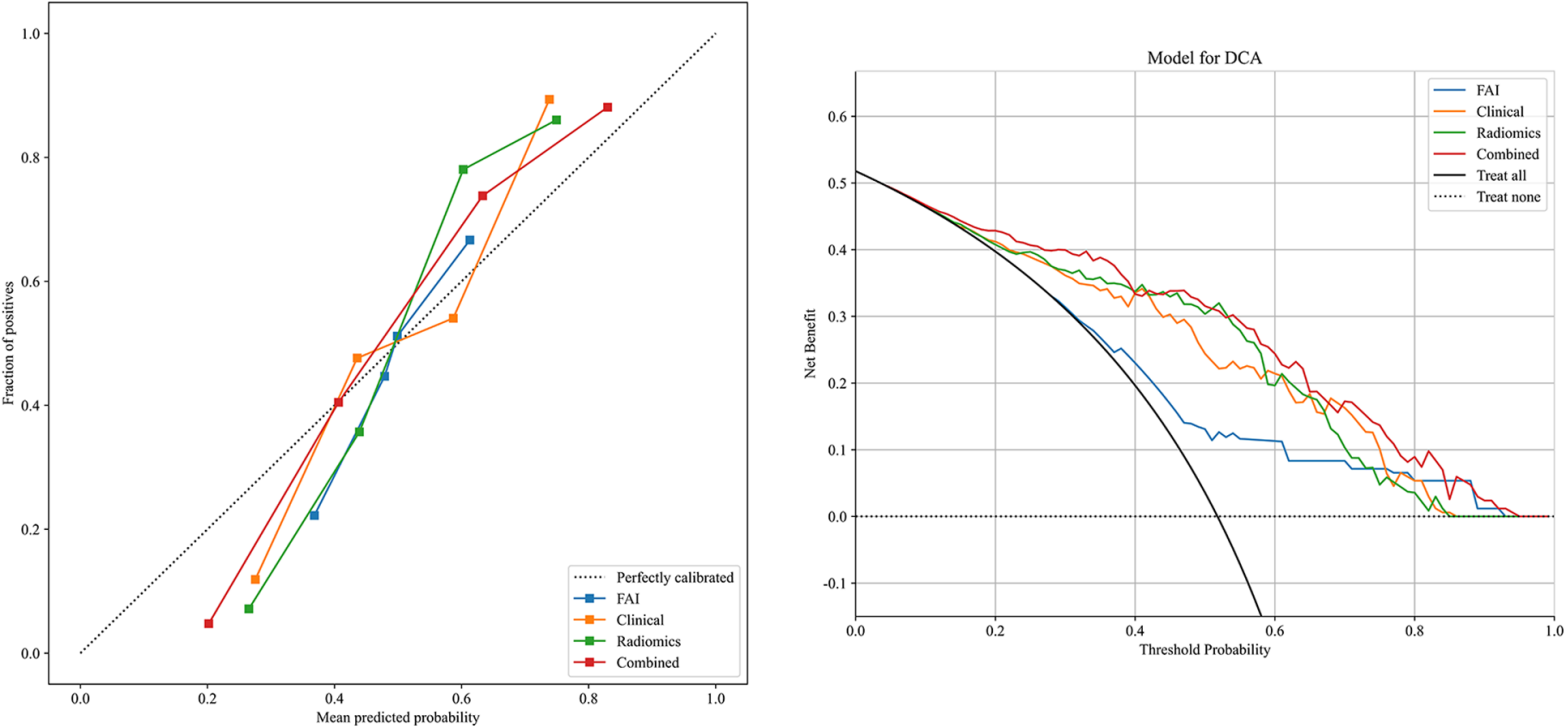
Decision curve analysis and calibration curves of the FAI model (blue line), clinical model (orange line), radiomics model (green line) and combined model (red line).

ROC curves for the four models were plotted in both the training and testing cohorts (see Figure 4), graphically representing the diagnostic performance of each model. Compared to the clinical feature model (AUC = 0.81 [95% CI: 0.728–0.882], AUC = 0.67 [95% CI: 0.526–0.812]) and the radiomics model (AUC = 0.71 [95% CI: 0.614–0.809], AUC = 0.54 [95% CI: 0.348–0.733]), the integrated model demonstrated superior discriminatory capacity (AUC = 0.83 [95% CI: 0.750–0.913], AUC = 0.71 [95% CI: 0.539–0.871]). The FAI model, constructed solely based on FAI values (AUC = 0.83 [95% CI: 0.750–0.913], AUC = 0.71 [95% CI: 0.539–0.871]), showed significantly inferior performance in distinguishing between stable and unstable angina compared to the other three models. The diagnostic abilities of the four models are presented through calculated accuracy, specificity, sensitivity, positive predictive value, and negative predictive value (Table 3). Calibration curves demonstrated good agreement between predictions and observations for the four models in differentiating UA from SA (see Figure 5). Decision curve analysis for the four models is shown in Figure 5, assessing whether the models provide a high net benefit for patients with angina. DCA indicates that, in distinguishing UA from SA, the overall net benefit of the integrated model is superior to that of the FAI model, clinical model, and radiomics model across most reasonable threshold ranges.

**Table 3:** Predictive ability of all models.

Model	Training cohort						Validation cohort					
	AUC (95% CI)	SPE	SEN	ACC	PPV	NPV	AUC (95% CI)	SPE	SEN	ACC	PPV	NPV
FAI	0.68 (0.60–0.76)	0.98	0.24	0.60	0.92	0.55	0.51 (0.38–0.64)	0.74	0.21	0.50	0.41	0.53
Clinical	0.84 (0.78–0.90)	0.63	0.89	0.76	0.72	0.84	0.67 (0.55–0.80)	0.74	0.61	0.68	0.67	0.69
Radiomics	0.85 (0.80–0.91)	0.82	0.79	0.80	0.82	0.79	0.73 (0.60–0.85)	0.85	0.67	0.76	0.79	0.75
Combined	0.87 (0.82–0.92)	0.79	0.83	0.81	0.81	0.81	0.74 (0.63–0.86)	0.59	0.79	0.68	0.62	0.77

FAI, fat attenuation index; AUC, area under curve; 95% CI, 95% confidence interval; SPE, specificity; SEN, sensitivity; ACC, accuracy; PPV, positive predictive value; NPV, negative predictive valve.

## 4 Discussion

Recent studies have indicated that inflammation, fibrosis, and microvascular remodeling are three principal contributors to the formation of coronary atherosclerosis (30). Consequently, an increasing number of researchers are attempting to identify a novel approach for the diagnosis and classification of coronary artery diseaseby focusing on these areas. With angina patients constituting a significant proportion of those with CAD (3, 28, 31), the ability to rapidly and accurately diagnose and differentiate between SA and UA is critical for stratifying subsequent risk management and selecting appropriate treatment strategies. SA typically results from fixed coronary atherosclerotic plaque obstruction, causing myocardial ischemic symptoms due to a relative constancy in myocardial blood supply, which can still maintain a supply-demand balance at rest. In contrast, UA is characterized by a range of clinical symptoms due to arterial plaque rupture or erosion, accompanied by varying degrees of surface thrombus formation, vasospasm, and distal embolism (7). Clinically, angina patients are broadly classified and initially diagnosed based on parameters such as the frequency and intensity of chest pain episodes, electrocardiogram results, and laboratory indicators. However, these changes are often the result of complex interactions within inflammatory responses, and conventional circulating inflammatory markers (e.g., high-sensitivity C-reactive protein and proinflammatory cytokines) lack specificity in identifying coronary inflammation (22). Furthermore, the invasiveness of coronary angiography limits its practical application. Therefore, this study utilizes the novel technique of radiomics, combining extracted imaging features with clinical features to construct an integrated model, in hopes that this model will enhance the diagnostic and discriminatory capabilities for CAD in clinical practice. Coronary CT angiography remains the preferred non-invasive imaging modality for diagnosing CAD, capable of evaluating coronary artery plaque status, degree of narrowing, and hemodynamic factors such as fractional flow reserve (6).

Pericoronary adipose tissue is the tissue in closest contact with the coronary artery wall and engages in bidirectional inflammatory signaling with it. PCAT attenuation is negatively correlated with histological adipocyte size and differentiation stage; higher PCAT attenuation indicates smaller adipocytes with lower fat content, shifting from a lipid-rich/water-poor phase in nearby non-diseased vessels to a lipid-poor/water-rich phase in inflamed vessels (9, 30). The Fat Attenuation Index can indirectly reflect coronary inflammation by capturing the average density values of PCAT. However, current CT density-based measurement methods reveal only the average voxel size of PCAT without considering the complex spatial relationships between voxels (11). Thus, the present study employs an analytical approach based on PCAT radiomic features and incorporates machine learning to automatically extract high-dimensional spatial quantitative features that are not discernible to the naked eye. This approach aims to build models that compensate for the limitations of pericoronary FAI values. Radiomics, succinctly put, is a method that extracts the most relevant quantitative features from large datasets of medical images to quantify phenotypic characteristics of lesions (32). It overcomes the limitations of subjective visual analysis, obtaining more comprehensive imaging information from each image to detect lesions, assist clinical diagnosis, and evaluate treatment effects. In this experiment, we segmented images of regions of interest in the three main coronary arteries and from their PCAT, extracted and selected the optimal 12 radiomic features. These features quantified variations in the size and shape of the coronary arteries in three-dimensional imaging datasets. Furthermore, we utilized these imaging feature values to construct corresponding machine learning models, which demonstrated robust performance in distinguishing between UA and SA patients. Among the myriad of risk factors associated with the progression of coronary heart disease (CHD), the atherogenic role of low-density lipoprotein is the most well-established. Genetic polymorphisms that regulate cholesterol transport and LDL metabolism are associated with CHD risk, and extensive large-scale observational cohort studies from around the world have shown a strong graded relationship between LDL-C levels and CHD risk (33). On the other hand, the cholesterol content of LDL particles is variable, and an increase in LDL-C levels can lead to the accumulation of lipids in blood vessels, accelerated plaque formation, and subsequent narrowing of the coronary arteries (34). The most commonly used anthropometric tool for assessing relative weight and classifying obesity is the Body Mass Index. A higher BMI indicates greater obesity, and the chronic accumulation of excess fat can lead to various metabolic changes, increasing the prevalence of cardiovascular disease risk factors and affecting systems that regulate vascular inflammation (31). Beyond serving as an independent cardiovascular disease risk factor, a high BMI status can also promote changes in other intermediate risk factors, such as dyslipidemia, hypertension, impaired glucose tolerance, inflammatory states, obstructive sleep apnea/hypopnea, and a prothrombotic state (35).

Studies have shown that age has a higher correlation with the risk of cardiovascular disease events in men than any other factor and is second only to hypertension in women (35–37). Scholars have further discovered that patients under 65 experience more frequent episodes of chest pain, whereas the frequency tends to decrease in patients over 65 (38). However, with increasing age, mortality rates also rise. This leads patients to disregard the increased risk that comes with age due to the alleviation of chest pain symptoms. In exploring the deeper causes of age and CHD, some have proposed the telomere theory—they suggest that telomere shortening is an indicator of the aging process, which can lead to atherosclerosis and thus cardiovascular disease (39). As age increases, telomeres become shorter, while a more balanced diet in CHD patients may lengthen telomeres (39). Our experiment also confirmed that age, LDL, and BMI can indeed make accurate diagnoses of unstable angina, and the clinical model established accordingly also has strong clinical performance. Finally, we combined the selected radiological features with the aforementioned three clinical features to create an integrated model nomogram, which significantly improved diagnosis and differentiation of unstable angina compared to traditional methods.

While our study demonstrates the potential of radiomics analysis of PCAT for distinguishing between UA and SA, it is important to acknowledge certain limitations inherent to the field of radiomics itself. Feature extraction in radiomics can vary significantly depending on imaging parameters (such as resolution, contrast, and modality) and the choice of segmentation algorithms, which may introduce variability into the results. This variability underscores the importance of standardizing imaging protocols and segmentation methods to enhance reproducibility. Additionally, as radiomics is a relatively new and evolving field, reproducibility and standardization across different clinical settings remain significant challenges. Differences in imaging equipment, software, and processing pipelines can impact the transferability of radiomics-based models, limiting their generalizability. To address these issues, future studies should aim to establish standardized protocols and validate models across multi-center cohorts to ensure robustness and reliability in diverse clinical environments.

This study does have certain limitations. Firstly, it is important to note that this study was conducted at a single center with a retrospective design, which may introduce selection bias. Such bias could lead to a lack of representativeness in the sample, potentially impacting the generalizability of the findings. Specifically, since the study was conducted in a specific hospital, the sample may not fully reflect the characteristics and clinical presentations of a broader population. This limitation could affect the external validity of our results, particularly in different geographical or healthcare settings. Future research should consider multi-center designs to comprehensively evaluate the applicability of PCAT radiomics across diverse populations. Additionally, prospective studies are needed to further validate our findings and minimize potential biases associated with retrospective analyses. Secondly, all patient images were acquired using the same CT scanner and settings. While image acquisition, reconstruction, and analysis can affect the reproducibility of radiomic features, no studies have investigated how these settings might influence radiomic parameters, therefore our model will require validation across different CT scanners in the future (40). Additionally, the number of PCAT radiomic features extracted through an entirely automatic process was limited; future work will incorporate more comprehensive feature extraction using deep learning models to better assist in the clinical identification of patients with early perimenopausal coronary heart disease. Lastly, some important clinical indices, such as C-reactive protein levels and electrocardiogram indicators, were not included in the clinical model, and their impact on the model's performance will need to be investigated in future research.

## 5 Conclusion

In summary, the PCAT radiomics model based on coronary CT can effectively identify unstable angina from stable angina. By combining radiomics characteristics and clinical risk factors, the integrated model can further improve the diagnosis and differential ability of unstable angina.

## Data Availability

The original contributions presented in the study are included in the article/Supplementary Material, further inquiries can be directed to the corresponding author.
